# Association between Prognostic Nutritional Index and Contrast-Associated Acute Kidney Injury in Patients Complicated with Chronic Kidney Disease and Coronary Artery Disease

**DOI:** 10.1155/2021/2274430

**Published:** 2021-07-05

**Authors:** Xiaoli Dong, Bo Wang, Shiqun Chen, Jin Liu, Yu Xia, Shouhong Wang, Bin Li, Sheng Wang, Ming Ying, Huanqiang Li, Ziling Mai, Yongquan Yang, Jiyan Chen, Yong Liu, Tiehe Qin, Ning Tan

**Affiliations:** ^1^The Second School of Clinical Medicine, Southern Medical University, Guangzhou 510515, China; ^2^Department of Cardiology, Guangdong Provincial Key Laboratory of Coronary Heart Disease Prevention, Guangdong Cardiovascular Institute, Guangdong Provincial People's Hospital, Guangdong Academy of Medical Sciences, Guangzhou 510080, China; ^3^Department of Cardiology, Hainan General Hospital, Hainan Affiliated Hospital of Hainan Medical University, Haikou 570311, China; ^4^Department of Cardiology, Qingyuan Hospital of Traditional Chinese Medicine Affiliated to Guangzhou University of Traditional Chinese Medicine, Qingyuan 511500, China; ^5^Department of Critical Care Medicine, Guangdong Provincial Geriatrics Institute, Guangdong Provincial People's Hospital, Guangdong Academy of Medical Sciences, Guangzhou 510080, China; ^6^Guangdong Provincial People's Hospital, School of Medicine, South China University of Technology, Guangzhou 510100, China

## Abstract

**Background:**

Contrast-associated acute kidney injury (CA-AKI) is a major adverse effect of coronary angiography (CAG). Patients with chronic kidney disease (CKD) and coronary artery disease (CAD) are at high risk of CA-AKI. This study aimed to investigate the association between prognostic nutritional index (PNI) and CA-AKI in this high-risk population.

**Methods:**

This study enrolled a total of 4,391 patients. CA-AKI was defined as a serum creatinine increase ≥0.3 mg/dL or 50% from baseline within the first 48 hours following CAG. The PNI was calculated upon hospital admission: serum albumin (g/L) + 5 × total lymphocyte count (10^9^/L). PNI was analysed from the high level to low level as a continuous variable and categorical variable which was divided into four groups by quartile. Restricted cubic splines and logistic regression were applied.

**Results:**

Overall, 13.09% (575/4391) of patients developed CA-AKI. PNI score was significantly lower in patients with CA-AKI than that in patients without CA-AKI (*P* < 0.01). The relationship between PNI score and CA-AKI was linear. A logistic regression model revealed that decreased PNI score was associated with increased risk of CA-AKI [per 1-point decrement; adjusted OR = 1.08, 95% CI, 1.05–1.09; compared with Quartile 1 (PNI ≥ 46.30), Quartile 4 (PNI < 37.90), adjusted OR = 1.88, 95% CI: 1.41–2.51; and Quartile 3 (37.90 ≤ PNI < 42.15), adjusted OR = 1.37, 95% CI: 1.02–1.84].

**Conclusion:**

Our study indicated a negative linear relationship between PNI score and CA-AKI in patients undergoing CAG complicated with CKD and CAD. It suggested that malnutrition is associated with increased risk of CA-AKI in this population.

## 1. Introduction

Contrast-associated acute kidney injury (CA-AKI) is the third largest cause of hospital-acquired kidney injury which is related to prolonged hospital stay and poor long-term prognosis [1, 2]. Chronic kidney disease (CKD) is a major risk factor for CA-AKI [3, 4]. Multiple risk factors associated with the development of CA-AKI are irreversible, such as age, diabetes, and heart failure [3]. Thus, identifying a potentially reversible risk is necessary.

It was demonstrated that malnutrition was common in patients with coronary artery disease (CAD) [5], and malnutrition was closely related to the occurrence of acute kidney injury (AKI) in hospitalized patients [6–8]. The prognostic nutritional index (PNI) is a screening tool for the nutritional status and has been described as a simple and objective indicator [9]. A previous study found an association between PNI and AKI in patients undergoing coronary artery bypass grafting and admitted in the coronary-care unit [8, 10]. Patients with CKD and CAD were at high risk of CA-AKI. However, no study investigated the association between PNI and CA-AKI among this population.

Therefore, the current study intends to explore the relationship between PNI and development of CA-AKI in patients with CKD and CAD undergoing coronary angiography (CAG).

## 2. Method

### 2.1. Study Design and Participants

**Figure 1:**
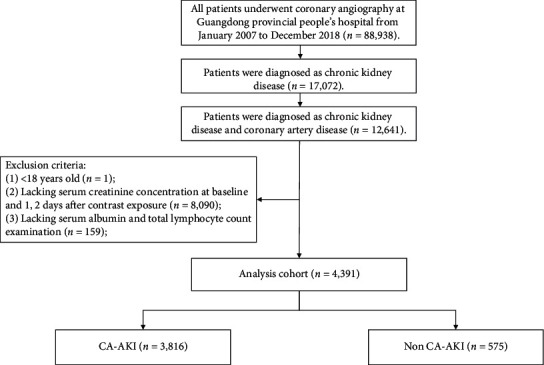
Study flow chart.

This retrospective observational study was processed using data from the Cardiorenal ImprovemeNt (CIN) study which was conducted in the largest cardiovascular centre in South China (Guangdong Provincial People's Hospital, China, Clinicaltrials.gov https://clinicaltrials.gov/ct2/show/NCT04407936). The baseline information including demographics, laboratory test results, mortality, and other clinical information was extracted from the electronic clinical management records system of Guangdong Provincial People's Hospital from January 2007 to December 2018. The follow-up information was retrieved from the Guangdong Public Security System and matched to the electronic Clinical Management System of the Guangdong Provincial People's Hospital records. Senior cardiologists were responsible for the data quality control and periodical data verification. All patients undergoing CAG between January 1, 2007, and December 31 were screened. During this period, there were 88,938 patients undergoing CAG and 12,641 patients were diagnosed as CKD and CAD. We excluded patients (1) <18 years of age (*n* = 1); (2) lacking serum creatinine concentration at baseline and 1, 2 days after contrast agent exposure (*n* = 8,090); and (3) lacking serum albumin, total lymphocyte count examination (*n* = 159) (Figure 1). The study protocol conformed to the principles outlined in the Declaration of Helsinki and was approved by the Guangdong Provincial People's Hospital ethics committee (No. GDREC2019555H[R1]).

### 2.2. Endpoint and Definition

The primary endpoint of this study was CA-AKI which was defined as an increase in SCr by 0.5 mg/dl or 25% within the first 48 h after the procedure [11]. PNI score was a screening tool to assess the nutritional status of hospitalized patients [9]. It was calculated by serum albumin and total lymphocyte count using the formula 10 × serum albumin (g/dl) + 5 × total lymphocyte count (10^9^/L). The formula for estimated glomerular filtration rate (eGFR) used was the Modification of Diet in Renal Disease (MDRD) formula (186 × SCr (mg/dL)^−1.154^ × age^−0.203^ × (0.742 for women) [12]. CKD was defined as eGFR ≤60 mL/min/1.73 m^2^ [13, 14]. CAD was confirmed by CAG and discriminated according to the 10th Revision Codes of the International Classification of Diseases. Comorbidities included acute myocardial infarction (AMI), diabetes mellitus, hypertension, congestive heart failure (CHF), and anemia. CHF was defined as New York Heart Association (NYHA) class > 2 or Killip class > 1 [3]. Anemia was defined as haematocrit <39% for men and haematocrit <36% for women, according to the World Health Organization criteria [15].

### 2.3. Statistical Analysis

Descriptive statistics for continuous variables with normal distribution and abnormal distribution are reported as mean (SD) and median (interquartile range [IQR]). Categorical variables are reported as numbers (percentages). Continuous variables with normal distribution were compared using independent samples Student's *t*-test. Differences between categorical variables were compared using the chi-square test. The independent associations between the CA-AKI and outcomes were assessed with a logistic regression model and expressed as odds ratio (OR) with 95% confidence interval (CI). Covariates in the model included age, male, AMI, hypertension, diabetes mellitus, CHF, anemia, PCI, and contrast media volume. Statistical analyses were performed using SR software, version 3.6.3 (R Foundation for Statistical Computing). All *P* values <0.05 were considered statistically significant.

## 3. Result

A total of 4,391 patients with CKD and CAD on admission were analysed in this study. Patients were split into two groups according to the presence or absence of CA-AKI. The mean age was 68.71 ± 9.95 years, and 73.38% patients were male.

**Figure 2:**
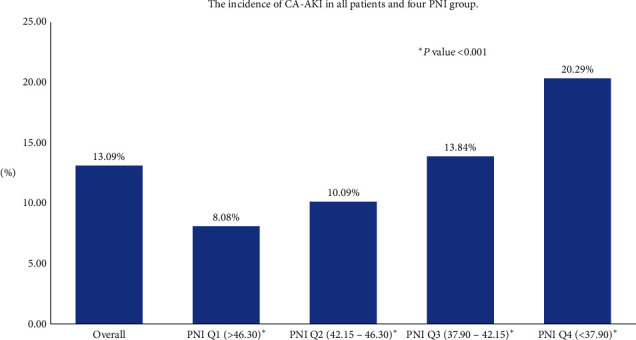
The incidence of CA-AKI in all patients and four PNI groups.

In the cohort, patients who developed AKI were elder and exhibited more comorbidities (diabetes mellitus, anemia, and CHF) and lower levels of lymphocyte, serum albumin, eGFR, and haematocrit compared with those who did not develop AKI. However, the sex distribution, PCI prevalence rate, PNI score, total cholesterol, high-density lipoprotein cholesterol (HDL-C), and low-density lipoprotein cholesterol (LDL-C) were comparable between two groups. More details of the baseline characteristics among enrolled patients are shown in Table 1. The percentage of people with CA-AKI accounted for 13.09% (*n* = 575) in all patients. The incidence of CA-AKI in four PNI groups which was divided by quartile from high to low was 8.08%, 10.09%, 13.84%, and 20.29%, respectively (Figure 2).

**Figure 3:**
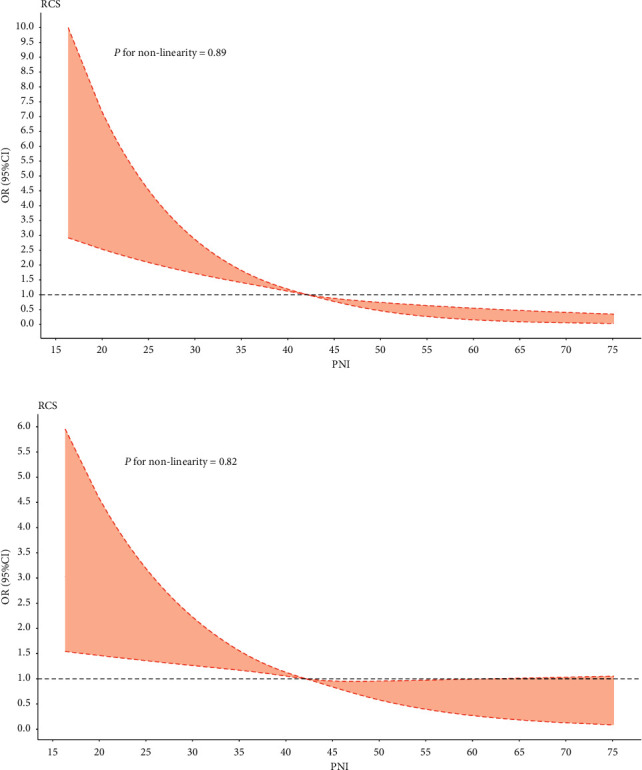
Restricted spline curve of the PNI score odds ratio for CA-AKI. (a) The restrict spline curve of the univariate cox model. (b) The restrict spline curve of the multivariate logistic model. ^*∗*^Adjusted for age, sex, acute myocardial infarction, hypertension, diabetes mellitus, congestive heart failure, anemia, PCI, and contrast media volume.

**Figure 4:**
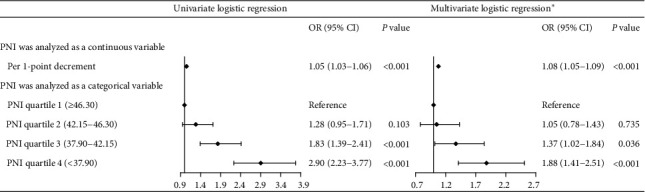
Univariate and multivariate logistic regression for CA-AKI. ^*∗*^Adjusted for age, sex, acute myocardial infarction, hypertension, diabetes mellitus, congestive heart failure, anemia, PCI, and contrast media volume.

There is a linear relation between PNI score and CA-AKI according to univariate and multivariate logistic models of restricted cubic splines (*P* for nonlinearity was 0.89 and 0.82, respectively, Figures 3(a) and 3(b)). A decreased PNI value was associated with an increased risk of CA-AKI. The result of multivariate logistic regression model showed that PNI value was associated with the occurrence of CA-AKI in patients with CKD and CAD after adjustment for clinical variables (Figure 4). When PNI was analysed as a continuous variable, lower PNI value was associated with the development of AKI (per 1-point decrement; adjusted OR = 1.08; 95% CI, 1.05–1.09) in multivariable regression. PNI was also analysed as a categorical variable and divided into four groups by quartile from high to low in the multivariable model. The lowest two groups of quartile in PNI were associated with 88% and 37% increased risk of CA-AKI compared with the highest quartile in all participants [compared with Quartile 1 (PNI ≥ 46.30), Quartile 4 (PNI < 37.90), adjusted OR = 1.88, 95% CI:1.41–2.51; and Quartile 3 (37.90 ≤ PNI < 42.15), adjusted OR = 1.37, 95% CI: 1.02–1.84].

## 4. Discussion

To our knowledge, this is the first study to analyse the association of PNI with CA-AKI in a large cohort among CKD patients complicated with CAD. Our data showed that low PNI score representing malnutrition is associated with increased risk of CA-AKI in the study population.

Malnutrition is common in patients with AKI and affects the occurrence and development of AKI independently of nonnutritional factors, increasing hospital death, complications, and medical costs [16–18]. In an observational study which recruited 6,444 patients from the Medical Information Mart for Intensive Care (MIMIC) III database and 412 patients from Zhongnan Hospital of Wuhan University conducted by Hu et al., the results demonstrated that PNI value was an independent predictor of AKI in patients within the coronary-care unit [8]. There are some similarities between this study and ours. Both studies intended to evaluate the association of PNI score with AKI. All the results indicated that decreased PNI level which represents malnutrition was related to increased risk of AKI. In addition, several other studies have also found that malnutrition is correlated to a rising risk of AKI. In a retrospective study enrolling hospital admissions, malnutrition assessed by Nutritional Risk Screening 2002 (NRS-2002) was strongly associated with the occurrence of AKI and worsened the prognosis [7]. Piggott et al. even found that malnutrition was independently associated with the occurrence of AKI in neonates (younger than 30 days) who underwent congenital heart surgery requiring cardiopulmonary bypass [6].

Serum albumin concentration and total lymphocyte count, as a component of PNI score which screens nutritional status, have also been shown to be related to the occurrence and development of CA-AKI. Several studies have demonstrated that low plasma albumin level (<35 g/L) is an independent risk factor for CA-AKI [19] and is closely related to poor prognosis in CAD [20]. Yu et al. reported that patients with low plasma albumin level (<35 g/L) had a high incidence of AKI in the hospital [21]. A meta-analysis conducted by Liu et al. indicated that hypoalbuminemia was independently associated with the occurrence of CA-AKI and increased the risk of CA-AKI by 1.59 times [22]. Previous studies also found that low lymphocyte count was also associated with an increased risk of CA-AKI. The result from a meta-analysis showed that on-admission platelet-to-lymphocyte ratio (PLR) level in the CA-AKI group is significantly higher than that of the non-CA-AKI group [23]. A retrospective study completed by Zorlu and Koseoglu demonstrated that platelet volume-to-lymphocyte ratio (MPVLR) was independently related to CA-AKI [24]. In summary, patients who developed CA-AKI had significantly higher volume-to-lymphocyte ratio (MPVLR), neutrophil-to-lymphocyte ratio (NLR), and platelet-to-lymphocyte ratio (PLR). This meant that patients with CA-AKI have lower lymphocyte count than patients without CA-AKI.

Malnutrition is defined as any nutritional imbalance, which is a major complication in hospitalized patients. The prevalence of malnutrition is 15–80% in hospitalized patients, while only 3% could be diagnosed and treated [25, 26]. It has been reported that over 40% of CKD patients are undernourished. Uraemia and dialysis can impact the nutritional status of patients with kidney disease [27]. The pathophysiology of CA-AKI may be relevant to inflammation, oxidative stress, and renal vasoconstriction [1, 4]. Albumin is the most abundant circulating protein and plays an important role in anti-inflammatory, antioxidant, anticoagulant, and antiplatelet aggregation activities [28, 29]. Lymphocytes play a major role in regulating the immune system [30], and inflammation enhances lymphocytic apoptosis [31]. Decreased albumin synthesis, increased catabolism, and aggravated inflammation contribute to malnutrition. These mechanisms may also accelerate the occurrence of CA-AKI.

Our findings strongly support the need for physicians to integrate the identification of malnutrition among patients with CKD and CAD before CAG in their daily practice. This may improve the risk stratification and may guide prevention of CA-AKI. With this easily calculable index which uses only 2 laboratory values, malnutrition can be screened timely. Patients at high risk of CA-AKI who might benefit from tailored nutritional supplements to reduce CA-AKI risk even improve their prognosis.

### 4.1. Limitation

We are focusing on the high-risk patients who need more attention from clinicians. However, we must acknowledge there were some limitations in this study. Firstly, this study is a single-centre retrospective study, but the enrolled patients came from the largest cardiac intervention centre in South China, which was representative and convincing in the sample and the study quality control. Secondly, the generalizability of our results is limited to the Chinese population without the consideration of other races, but our findings could be extrapolated to other countries with similar health systems and populations. Thirdly, there were limited data on the included patients, without information about inflammation markers such as CRP, which might help us assess the association between PNI score and CA-AKI comprehensively. However, PNI score was calculated by serum albumin concentration and lymphocyte count which decreased due to the effects of inflammation.

## 5. Conclusions

In conclusion, there existed a negative linear relationship between PNI score and CA-AKI in patients undergoing CAG complicated with CKD and CAD. The current study indicated that PNI value could serve as a preprocedural predictor for the development of CA-AKI in CKD patients complicated with CAD who received CAG. These patients with low PNI score which represented as malnutrition should be paid more attention before presentation CAG.

## Figures and Tables

**Table 1:** Baseline characteristics.

Characteristic^*∗*^	Non-CA-AKI	CA-AKI	*P* value
	(*n* = 3816)	(*n* = 575)	
*Demographic characteristics*			
Age (year)	68.47 (9.92)	70.32 (10.05)	<0.001
Age ≥ 75 (*n* (%))	1139 (29.85)	220 (38.26)	<0.001
Male (*n* (%))	2807 (73.56)	415 (72.17)	0.52

*Coexisting conditions*			
AMI (*n* (%))	890 (23.34)	155 (26.96)	0.07
Hypertension (*n* (%))	2781 (72.93)	425 (73.91)	0.66
Diabetes mellitus (*n* (%))	1501 (39.37)	268 (46.61)	0.001
Anemia (*n* (%))	2138 (56.03)	424 (73.74)	<0.001
CHF (*n* (%))	823 (21.59)	232 (40.35)	<0.001
Hypoalbuminemia (*n* (%))	2119 (55.53)	386 (67.13)	<0.001

*Procedure*			
PCI (*n* (%))	2942 (77.10)	424 (73.74)	0.09
Contrast media volume (100 mL)	1.54 (0.84)	1.60 (0.89)	0.10

*Laboratory examination*			
Haematocrit (%)	0.37 (0.06)	0.34 (0.06)	<0.001
Lymphocyte (10^9^/L)	1.67 (0.68)	1.44 (0.62)	<0.001
Total cholesterol (mmol/L)	4.42 (1.21)	4.43 (1.26)	0.90
HDL-C (mmol/L)	0.95 (0.26)	0.96 (0.26)	0.40
LDL-C (mmol/L)	2.69 (0.96)	2.70 (0.95)	0.87
Albumin (g/L)	34.05 (4.67)	32.39 (5.25)	<0.001
eGFR (mL/min/1.73 m^2^)	43.14 (14.07)	35.92 (15.26)	<0.001
Serum creatinine (mmol/L)	174.28 (144.38)	219.46 (178.54)	<0.001
PNI score	42.41 (6.25)	39.59 (6.45)	<0.001

*Medicine*			
RASi (*n* (%))	1655 (44.83)	144 (28.74)	<0.001
*β*-blocker (*n* (%))	2970 (80.44)	378 (75.45)	0.01
Statins (*n* (%))	3476 (94.15)	458 (91.42)	0.02

^*∗*^Data are presented as the mean value (standard deviation) or number of participants (percentage). Abbreviations: AMI, acute myocardial infarction; CHF, congestive heart failure; PCI, percutaneous coronary intervention; HDL-C, high-density lipoprotein cholesterol; LDL-C, low-density lipoprotein cholesterol; PNI score, prognostic nutritional index score; RASi, renin angiotensin system inhibitor.

## Data Availability

The datasets analysed during the current study will be available from the corresponding author on reasonable request when the study is finished.

## Abbreviations

CA-AKI: Contrast-associated acute kidney injury
CKD: Chronic kidney disease
CAD: Coronary artery disease
PNI: Prognostic nutritional index
CAG: Coronary angiography
PCI: Percutaneous coronary intervention
AMI: Previous acute myocardial infarction
CHF: Congestive heart failure
OR: Hazard ratio
CI: Confidence interval.

## References

[1] McCullough P. A., Choi J. P., Feghali G. A. (2016). Contrast-induced acute kidney injury. *Journal of the American College of Cardiology*.

[2] Sun G., Chen P., Wang K. (2019). Contrast-induced nephropathy and long-term mortality after percutaneous coronary intervention in patients with acute myocardial infarction. *Angiology*.

[3] Mehran R., Aymong E., Nikolsky E. (2004). A simple risk score for prediction of contrast-induced nephropathy after percutaneous coronary intervention development and initial validation. *Journal of the American College of Cardiology*.

[4] Mehran R., Dangas G. D., Weisbord S. D. (2019). Contrast-associated acute kidney injury. *New England Journal of Medicine*.

[5] Raposeiras Roubín S., Abu Assi E., Cespón Fernandez M. (2020). Prevalence and prognostic significance of malnutrition in patients with acute coronary syndrome. *Journal of the American College of Cardiology*.

[6] Piggott K. D., Liu A., Monczka J. (2018). Inadequate preoperative nutrition might be associated with acute kidney injury and greater illness severity postoperatively. *The Journal of Thoracic and Cardiovascular Surgery*.

[7] Li C., Xu L., Guan C. (2020). Malnutrition screening and acute kidney injury in hospitalised patients: a retrospective study over a 5-year period from China. *British Journal of Nutrition*.

[8] Hu Y., Cao Q., Wang H. (2021). Prognostic nutritional index predicts acute kidney injury and mortality of patients in the coronary care unit. *Experimental and Therapeutic Medicine*.

[9] Buzby G. P., Mullen J. L., Matthews D. C., Hobbs C. L., Rosato E. F. (1980). Prognostic nutritional index in gastrointestinal surgery. *The American Journal of Surgery*.

[10] Dolapoglu A., Avci E., Kiris T., Bugra O. (2019). The predictive value of the prognostic nutritional index for postoperative acute kidney injury in patients undergoing on-pump coronary bypass surgery. *Journal of Cardiothoracic Surgery*.

[11] Stacul F., van der Molen A. J., Reimer P. (2011). Contrast induced nephropathy: updated ESUR contrast media safety committee guidelines. *European Radiology*.

[12] Levey A. S., Bosch J. P., Lewis J. B., Greene T., Rogers N., Roth D. (1999). A more accurate method to estimate glomerular filtration rate from serum creatinine: a new prediction equation. *Annals of Internal Medicine*.

[13] National Kidney Foundation (2002). K/DOQI clinical practice guidelines for chronic kidney disease: evaluation, classification, and stratification. *American Journal of Kidney Diseases*.

[14] Manjunath G., Tighiouart H., Ibrahim H. (2003). Level of kidney function as a risk factor for atherosclerotic cardiovascular outcomes in the community. *Journal of the American College of Cardiology*.

[15] (1968). Nutritional anaemias. report of a WHO scientific group. *World Health Organization Technical Report Series*.

[16] Han S.-H., Han D.-S. (2012). Nutrition in patients on peritoneal dialysis. *Nature Reviews Nephrology*.

[17] Alp Ikizler T., Cano N. J., Franch H. (2013). Prevention and treatment of protein energy wasting in chronic kidney disease patients: a consensus statement by the international society of renal nutrition and metabolism. *Kidney International*.

[18] Keskin H. A., Kurtul A., Esenboğa K., Çiçek M. C., Katırcıoğlu S. F. (2020). Prognostic nutritional index predicts in-hospital mortality in patients with acute stanford type A aortic dissection. *Perfusion*.

[19] Murat S. N., Kurtul A., Yarlioglues M. (2015). Impact of serum albumin levels on contrast-induced acute kidney injury in patients with acute coronary syndromes treated with percutaneous coronary intervention. *Angiology*.

[20] Shaper A. G., Wannamethee S. G., Whincup P. H. (2004). Serum albumin and risk of stroke, coronary heart disease, and mortality: the role of cigarette smoking. *Journal of Clinical Epidemiology*.

[21] Yu M.-Y., Lee S. W., Baek S. H. (2017). Hypoalbuminemia at admission predicts the development of acute kidney injury in hospitalized patients: a retrospective cohort study. *PLoS One*.

[22] Liu L., Lun Z., Wang B. (2021). Predictive value of hypoalbuminemia for contrast-associated acute kidney injury: a systematic review and meta-analysis. *Angiology*.

[23] Kurtul A., Ornek E. (2019). Platelet to lymphocyte ratio in cardiovascular diseases: a systematic review. *Angiology*.

[24] Zorlu C., Koseoglu C. (2020). Comparison of the relationship between inflammatory markers and contrast-induced nephropathy in patients with acute coronary syndrome after coronary angiography. *Angiology*.

[25] Dewey R., Levings C. S., Timothy D. H. (1986). Novel recombinations in the maize mitochondrial genome produce a unique transcriptional unit in the Texas male-sterile cytoplasm. *Cell*.

[26] Hartz L. L. K., Stroup B. M., Bibelnieks T. A., Shockey C., Ney D. M. (2019). ThedaCare nutrition risk screen improves the identification of non-intensive care unit patients at risk for malnutrition compared with the nutrition risk screen 2002. *Journal of Parenteral and Enteral Nutrition*.

[27] Malone A., Hamilton C. (2013). The academy of nutrition and dietetics/the American society for parenteral and enteral nutrition consensus malnutrition characteristics. *Nutrition in Clinical Practice*.

[28] Arques S. (2018). Human serum albumin in cardiovascular diseases. *European Journal of Internal Medicine*.

[29] Soeters P. B., Wolfe R. R., Shenkin A. (2019). Hypoalbuminemia: pathogenesis and clinical significance. *Journal of Parenteral and Enteral Nutrition*.

[30] Weber C., Zernecke A., Libby P. (2008). The multifaceted contributions of leukocyte subsets to atherosclerosis: lessons from mouse models. *Nature Reviews Immunology*.

[31] Azab B., Zaher M., Weiserbs K. F. (2010). Usefulness of neutrophil to lymphocyte ratio in predicting short- and long-term mortality after non-ST-elevation myocardial infarction. *The American Journal of Cardiology*.

